# Supplementary material for: The interactome of intact mitochondria by cross-linking mass spectrometry provides evidence for coexisting respiratory supercomplexes
Source: Mol Cell Proteomics. 2017 Dec 8;17(2):216–32. doi: 10.1074/mcp.RA117.000470 (PMC5795388; doi:10.1074/mcp.RA117.000470)
Supplement: Supplemental Data [file supp_RA117.000470_133922_0_supp_23978_fzffwf.zip › spectra_annotation/mito_spectra_annotation/335-1-4-1-4-1.pdf]

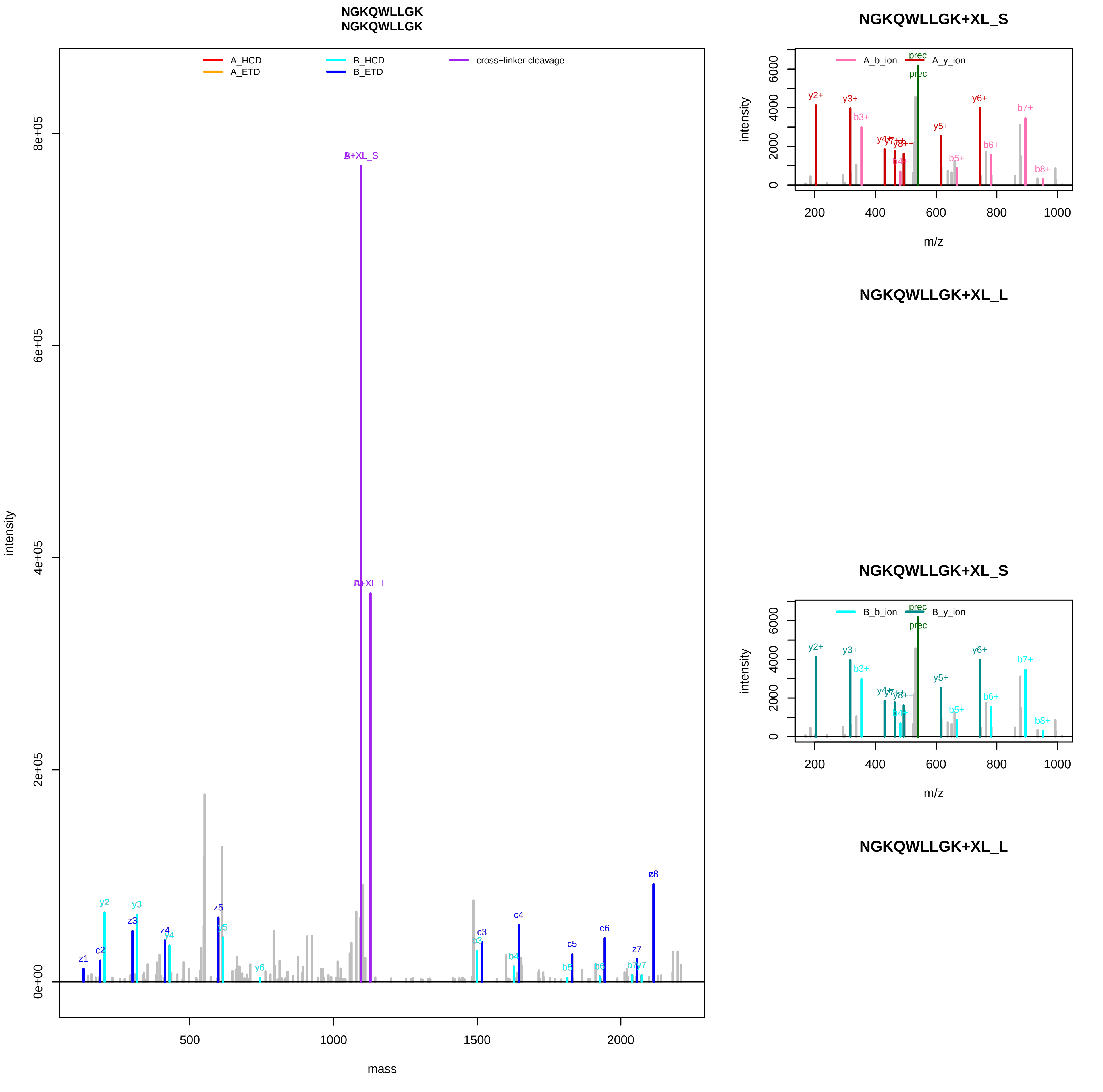
NGKQWLLGK
NGKQWLLGK
A_HCD
A_ETD
B_HCD
B_ETD
cross−linker cleavage
B+XL_S
B+XL_L
intensity
mass
z1
c2
y2
z3
y3
z4
y4
z5
y5
y6
b3
c3
b4
c4
b5
c5
b6
c6
b7
z7
y7
z8
NGKQWLLGK+XL_S
A_b_ion
A_y_ion
prec
y2+
y3+
b3+
y4+
y7++
y8++
b4+
y5+
b5+
y6+
b6+
b7+
b8+
m/z
NGKQWLLGK+XL_L
NGKQWLLGK+XL_S
B_b_ion
B_y_ion
NGKQWLLGK+XL_L
